# Exploring the interaction of activity limitations with context, systems, community and personal factors in accessing public health care services: A presentation of South African case studies

**DOI:** 10.4102/phcfm.v9i1.1166

**Published:** 2017-02-08

**Authors:** Gubela Mji, Stine H. Braathen, Richard Vergunst, Elsje Scheffler, Janis Kritzinger, Hasheem Mannan, Marguerite Schneider, Leslie Swartz, Surona Visagie

**Affiliations:** 1 Centre for Rehabilitation Studies, Stellenbosch University, South Africa; 2 SINTEF, Norway; 3 Psychology Department, Stellenbosch University, South Africa; 4 School of Nursing, Midwifery & Health Systems, Health Sciences Centre, University College Dublin, Ireland; 5 Alan J Flisher Centre for Public Mental Health, University of Cape Town, South Africa

## Abstract

**Background:**

There are many factors that influence access to public health services, such as the context people live in, the existing health services, and personal, cultural and community factors. People with disabilities (activity limitations), through their experience of health services, may offer a particular understanding of the performance of the health services, thus exposing health system limitations more clearly than perhaps any other health service user.

**Aim:**

This article explores how activity limitations interact with factors related to context, systems, community and personal factors in accessing public health care services in South Africa.

**Setting:**

We present four case studies of people with disabilities from four low-resource diverse contexts in South Africa (rural, semi-rural, farming community and peri-urban) to highlight challenges of access to health services experienced by people with activity limitations in a variety of contexts.

**Methods:**

One case study of a person with disabilities was chosen from each study setting to build evidence using an intensive qualitative case study methodology to elucidate individual and household experiences of challenges experienced by people with activity limitations when attempting to access public health services. In-depth interviews were used to collect data, using an interview guide. The analysis was conducted in the form of a thematic analysis using the interview topics as a starting point.

**Results:**

First, these four case studies demonstrate that equitable access to health services for people with activity limitations is influenced by a complex interplay of a variety of factors for a single individual in a particular context. Secondly, that while problems with access to public health services are experienced by everyone, people with activity limitations are affected in particular ways making them particularly vulnerable in using public health services.

**Conclusion:**

The revitalisation of primary health care and the introduction of national health insurance by the Health Department of South Africa open a window of opportunity for policy makers and policy implementers to revisit and address the areas of access to public health services for people with activity limitations.

## Introduction

This article explores how activity limitations interact with variables related to context, systems, community and personal factors in accessing public health care services in South Africa (SA). The findings in this article are based on data that were part of a larger EU-funded project, EquitAble, on access to health care for people with disabilities in four countries in Africa (Sudan, Malawi, Namibia and South Africa; www.equitableproject.org). We present four case studies of people with disabilities from four low-resource diverse contexts in SA (rural, semi-rural, farming community and peri-urban) to highlight challenges of access to health services experienced by people with activity limitations in a variety of contexts. These four case studies demonstrate that equitable access to health services for people with activity limitations is influenced by a complex interplay of a variety of factors for a single individual in a particular context.

There are many factors that influence access to health services, such as the context people live in, the existing health services, and personal, cultural and community factors.^1,2^ Accessible and equitable health services have four principal components: non-discrimination, physical accessibility, economic accessibility and information accessibility.^3^ While different vulnerable groups of people experience similar challenges with regard to equitable access to health care, in a context of poverty, there are also distinctive challenges experienced by each group.^4^ People with disabilities, through their experience of health services, may offer a particular understanding of the performance of the health services, thus exposing health system limitations more clearly than perhaps any other health service user.^5,6^ The importance of addressing both the health needs of vulnerable groups, such as people with disabilities in low-income countries, and the many challenges faced in meeting these needs for different categories of people, is increasingly being recognised.^5,2^ The Universal Declaration of Human Rights points to the fact that health care can neither be universal nor equitable if it is less accessible to some sectors of society.^7^

## Developing the conceptual framework: Interaction of activity limitations with context, systems, personal and community factors

The conceptual framework that we used for this paper is the one that is central to the International Classification for Functioning, Disability and Health (ICF).^8^ According to the ICF, disability is a complex and multidimensional phenomenon and reflects the interaction between features of the individual (e.g. personal factors, impairments, health condition and activity limitations) and factors external to the individual (e.g. cultural and social factors, the natural and built environment, policies, systems and services).^8^ Disability is measured on a continuum from optimal functioning to severe disability, with measures of disability reflecting this continuum by using a scale of responses from no difficulty through to inability to do an activity. The ICF further acknowledges that the majority of human beings experience some degree of disability during their life span and thus recognises that disability is a universal experience and not just one that happens to a minority of the population.^8,9^

International projects, such as Measuring Health and Disability in Europe^5^ and the Washington Group on disability statistics, have found that the continuum of activity limitations offers the most accurate scale to measure disability in self-reported and observational studies.^9,10^ In this article, we use people with disabilities and people with activity limitations interchangeably.

Different features of individuals and the context in which they live will determine whether these four elements relating to accessibility listed by the United Nations^3^ (see above) are realised or not. A number of factors, other than disability related ones, contribute to other sectors of the population also being vulnerable to inequitable access to health care.

These include the contexts in which people live (highly dispersed areas, displaced persons, in chronic poverty, or in areas with high inequalities) and the existing health system’s distribution of resources between different services, the emphasis on primary care, and the extent of service integration.^11^ The link between activity limitations and access is also influenced by personal factors (coping skills, gender, age and ethnicity) and community factors (cultural understandings of disability, extent of family support and opportunities for inclusion).

**FIGURE 1:**
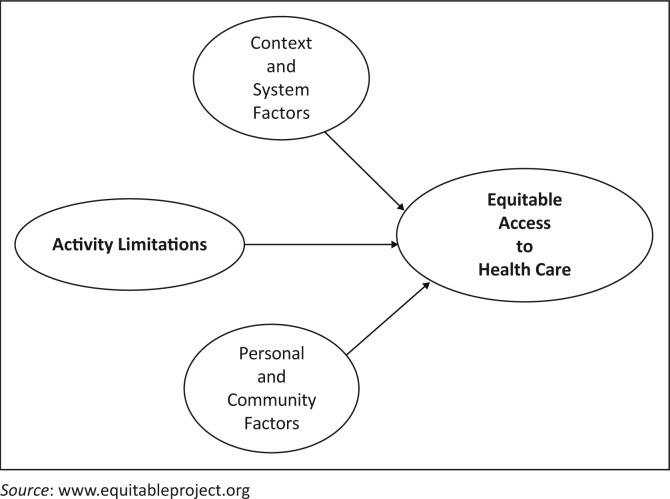
EquitAble conceptual framework.

The EquitAble project researchers developed a conceptual framework based on the ICF and the four components of accessible and equitable access to health care as set out by the United Nations Committee on Economic, Social and Cultural Rights.^12,8^ Each case study will be mapped against the equitable conceptual framework (see Figure 1) to see how activity limitations interact with the context, systems and personal and community factors.^8^ In this paper, we have replaced the term variables from the original equitable conceptual framework with factors. Although similar studies on health needs for vulnerable groups were done by Dixon-woods^2^, Lee et al.^13^ and Leight^14^, none of them explored how activity limitations or disability interact with these factors to influence access to public health services.

## Methodology

Four South African sites were purposively selected to depict the high levels of inequality that are found in SA. The sites were chosen within three provinces: Northern Cape, Eastern Cape and Western Cape. Findings presented in this paper came from data gathered during a descriptive, qualitative process that formed part of the EquitAble project. Respondents included people with disabilities identified through a snowballing approach using the local health facility, community leaders, and institutions for people with disabilities in the communities, health care service providers and community leaders or whoever was known by the researchers. While information in this paper came primarily from four case studies, it is supported by data from other sources of information from the qualitative data of the EquitAble project in each of the sites. The choice of one case study for each study setting was to build evidence using an intensive qualitative case study methodology to elucidate individual and household experiences of challenges experienced by people with activity limitations when attempting to access public health services. We argue that because of the diverse contexts in SA, a single case study for each context provides a range of different South African challenges.^15,16,17,18,19^

In-depth interviews were used to collect data, using an interview guide which included demographic detail, information on access and use of health services, family structure, support systems and economic status. This ensured that similar topics were covered across the four case studies but also allowing for interviewees to raise their own topics. The interviews were conducted in the preferred language of the participant, by a fieldworker that was trained in data collection techniques. Trained interpreters were used in instances where the interviewee’s preferred language was not the same as that of the fieldworker. Interviews took place in a locality where participants felt comfortable. The interviews were recorded, and the recordings transcribed and translated into English for the analysis. Researchers that were involved in the preparation of this manuscript were also involved in ensuring quality and reliability of data and data analysis.

The analysis was conducted in the form of a thematic analysis using the interview topics as a starting point, and also allowing the data to provide further themes not covered in the interview guide. The ATLAS.ti software programme was used for the analysis of the interviews. Information was obtained from a number of participants, but this article will focus on just one case study from each study site to give the most detailed narratives highlighting the complex interplay of factors for people with activity limitations when they attempt to access public health services in the areas where they live. As the data for the four case studies were drawn from the qualitative component of the EquitAble project, data for triangulation were drawn from brief analyses of other interviews, knowledge of the areas and findings from key informant interviews.

## Ethical considerations

Ethical clearance and approval for the project were obtained from the Committee for Human Research of Stellenbosch University, as well as from the relevant provincial department of health and city health authorities. Participation in the study was voluntary, and all participants gave their oral or written consent before data collection commenced. Researchers explained to participants that information would be kept anonymous and pseudonyms used in writing up of study findings.

## Presentation of the four case studies

This section presents a brief description of the four sites and biographical information on the four participants and their context related to accessing the public health care services. The information was obtained from a triangulation of a number of sources of data collected during the study period. These included the four case study participants/interviewees/respondents, other interviewees/respondents/participants, key informant interviews and observations of the different health care facilities.

### Gugulethu in the Western Cape province

Gugulethu is a small, densely populated (16 000 people per km^2^), predominantly Black Xhosa-speaking township in the eastern substructure of the City of Cape Town. The population is young, with 88% being under 50 years of age^19^ and 70% having not completed their high school education.^20^ Poverty, crime and substance abuse are rife in the community and homicide is the main cause of death (23.4%).^21,22^ At the time of data collection, large parts of the community did have access to electricity, cell phone connectivity and Internet access. There is also public transport available in the community in the form of trains, buses and taxis.

Public health care services in Gugulethu are provided by four clinics and one community health centre (CHC), 5 days a week from 7.30 am until 4.30 pm. Services in the four clinics are nurse-driven with occasional visits by doctors. A comprehensive multidisciplinary health care team performs services at the CHC.

### Xoliswa

Forty-year-old Xoliswa is a wheelchair user, who is also partially sighted. She works in sheltered employment as she has limited formal education and her primary income is a social grant. Xoliswa is single and lives with her family 2 km from the nearest clinic. She relies on others for assistance for most daily activities. According to Xoliswa, having access to appropriate health care is one of the most important determinants of her health. Most people living in poverty access clinics on foot, or in Xoliswa’s case, with her wheelchair. Although she is able to negotiate the streets to the clinic, inclement weather and criminal violence makes her reluctant to travel this way. Taxi operators are reluctant to take on passengers in wheelchairs, or they charge additional fares. Wheelchair-accessible public transport must be booked 7 days in advance, which is impractical when one is ill. Private taxis are not affordable to the poor.

Access to secondary and tertiary services is gained through referral from primary level only. Clinics optimise service delivery by grouping together people with similar medical needs, thus offering specific services at certain times only. According to Xoliswa, her limited mobility makes it difficult for her to be on time and sometimes result in her missing these services.

### Worcester in the Western Cape province

Worcester town, situated 96 km outside of Cape Town, is classified as a rural area within the demographics of the province.^23^ It is a predominantly Mixed-Race, Afrikaans-speaking community, which is well serviced in terms of sanitation, water supply and waste disposal, with 42% of the people employed in the Agriculture sector.^23^ The inhabitants of the two surrounding townships are poor and racially segregated, that is, one being predominantly Mixed-Race and the other Black. The surrounding farms are predominantly White-owned and employing Mixed-Race and Black labourers. Worcester has well-established institutions for people with hearing and visual disabilities. The catchment area for the local clinic (Worcester Community Day Centre) is wide, and users have to travel vast distances to access health care, and mobile outreach services are limited. There are smaller nurse-driven clinics in other towns in the district.

#### Jaco

Jaco has a hearing impairment and is an Afrikaans-speaking, middle-aged male, with a chronic heart condition and diabetes. With a junior secondary education, he is knowledgeable about his own health and the health facilities in the area. Orphaned as a child, Jaco was raised by his adoptive mother. They moved frequently and finally to a farm where he slept outside in a small tent ‘… like a dog …’ through the very cold winters. His health deteriorated and the welfare services moved Jaco to the National Institute for the Deaf in Worcester, where he still lives.

While on the farm, he was only able to access health services once a week when he could get a lift to town with the farmer’s wife. In an emergency, with no available ambulance vehicles, they phoned the police who sent a vehicle to transport him to the doctor in town. Jaco experiences the WCDC as always very full, with preferential treatment given to people collecting chronic medication.

Unlike people who are completely deaf, Jaco has some but limited hearing and can use oral language to ask for help; therefore, he feels confident enough to use the health services independently. His experience of the health service staff is that they are either unaware that he is hearing impaired, or that they do not know how to communicate effectively with him, even though his impairment is indicated on his file. Despite trouble communicating, he still feels that the doctors give him sufficient time during consultations.

At the time of data collection, Jaco was receiving a government grant of which 75% was spent on accommodation in the National Institute for the Deaf and the remaining amount for toiletries, a funeral policy and cell phone. This left very little for unexpected medical expenses like his broken hearing aid, which would cost approximately 85 ZAR just to courier to Cape Town to get fixed. Jaco does not have the resources available for medical insurance, thereby is restricted in his choice of medical facilities to the public health facilities.

### Fraserburg in the Northern Cape province

The Northern Cape province is sparsely populated with vast distances and poor quality (gravel) roads between towns and farms. It is the largest province in SA but with the smallest population, – 1.2 million people. Just under half of the population live in poverty, and 40% are unemployed.^24^ Many members of the community are dependent on social grants provided by the government as their only means of income

The Mixed-Race population group accounts for 51.6% of the province’s population followed by Blacks at 35.7% and Whites at 12.4%. Fraserburg is a rural town whose community is built around commercial farming. The closest secondary and tertiary hospitals are in Calvinia (some 200 km away) and Kimberley (some 500 km away), respectively. Fraserburg represents a low resourced rural community with wide inequities in available resources between different social groups in the community. At the time of data collection, large parts of the community did not have access to electricity, cell phone connectivity and Internet access. There was no public transport in the community. Alcohol and related disabilities (e.g. foetal alcohol syndrome) are significant problems in the area. A nurse-driven Community Health Care Centre (CHCC) delivers formal public health care services to the community. A doctor is available once a week.

#### Emily

At 73 years of age, Emily is still an active member of the community of Fraserburg. She is involved as a volunteer in the running of a community development coffee shop. Emily has a visual impairment as a result of a childhood disease, rendering it difficult to see even when wearing glasses and causing constant pain. She can do tasks such as cooking, but cannot see well enough to read, sew or use a cell phone independently.

Emily uses chronic medication for her eyes that is prescribed at a tertiary government hospital but is not available at the CHCC in town, the only government health care service provider in the community. Emily`s challenges with regard to accessing medication were not unique. According to Emily and confirmed by service providers, shortages of medication were often experienced at the CHCC. Emily’s husband is employed and she could thus afford the cost of both medical insurance and transport to pharmacies 200 km away. Emily expresses clearly her opinion about health care in Fraserburg and identified various issues that increase vulnerability in the community. These include the lack of a permanent doctor, challenges with regard to transport, road infrastructure and erratic ambulance service, and a shortage of nursing staff at the CHCC which causes long waiting times. In spite of this, she sees the nursing staff showing positive, supportive attitude, very hard working and trusted by patients. Another positive aspect described by Emily is an appropriate, suitable facility. Finally, she felt that district, provincial and national government departments do not care about the plight of the community of Fraserburg and complaints are not dealt with.

### Madwaleni in the Eastern Cape province

Madwaleni, in the Eastern Cape province, is a deeply rural, traditional Xhosa area with many inequities, poverty and high levels of unemployment. It is one of SA’s poorest districts.^11^ The area is characterised by rugged hills, rivers, forests, gravel roads, free running animals and grass-thatched rondavels scattered sporadically over the hills. Madwaleni Hospital, a 200-bed secondary hospital, serves approximately 30 villages with approximately 200 000 inhabitants. There are approximately 120 000 people with 89% unemployed. There is a scarcity of sewage systems, running water and electricity to the general Madwaleni community, and these are limited to the hospital and the local hotels. This rural area is defined by poor infrastructure, lack of basic service provision, low levels of literacy, high levels of unemployment, limited access to health care and education, high incidence of communicable diseases and high mortality rates.

The area is also served by eight nurse-run satellite clinics around Madwaleni Hospital. The hospital and the clinics experience severe staff shortage and high staff turn-over and are all located in areas accessed with poor quality gravel roads.

#### Thandie

Thandie is a 27-year-old single mother to a 15-month-old daughter. She has a Grade 5 education, is unemployed, her monthly income level being dependent on disability and child support grants (approximately 1200 ZAR and 200 ZAR), which she says is very low, to support her and her child’s needs. Early in her pregnancy she was diagnosed with tuberculosis of the spine resulting in paraplegia. Following her diagnosis, she started using a wheelchair and walker. She subsequently has had to move and stay with her grandmother who lives close to the road where taxi services to the hospital can be accessed more easily.

Thandie says that her family have not been very supportive of her situation, and she perceives her partner and father of her child who lives in Johannesburg, as no longer interested in her and having abandoned her and her child.

Thandie told us that every time she has to go to Madwaleni Hospital, usually for physiotherapy, she has to arrange transport the night before, and she still risks waiting for hours before the arranged transport arrives the following morning. The most convenient for her would be to hire a taxi for herself and her wheelchair, but this would cost 350 ZAR per visit, and she cannot afford that.

Occasionally she is referred to Bedford Hospital in Mthatha for follow-up and treatment. Although free, the ambulance leaves Madwaleni Hospital very early in the morning. To be on time, Thandie has to sleep the night before on the benches of the hospital and as the hospital does not provide beds for people to stay overnight unless they are admitted to the in-patient ward. This she feels is extremely undignified and uncomfortable.

## Findings and discussion

Four themes emerged from the participants’ experience of access to public health services. These were: structural and environmental factors, systemic factors, activity limitations, and personal and cultural factors. We also included a heading presenting our observation of participants’ responses with regard to their lack of critical appraising of the health systems they used. We end by presenting an adjusted equitable conceptual framework.

## Structural and environmental factors

Structural and environmental factors are factors such as distances from public health services, transport, poor roads and cost of transport. Three of the four case study participants live in an environment of poverty, making them more vulnerable to poor access to health care.^25,26^ In Gugulethu, Xoliswa must either pay additional transport costs for her wheelchair, or brave the weather and crime to go to the clinic, while in Madwaleni, Thandie is unemployed and dependent on grants. Thandie has no choice but to pay for transport to get her to the health facility which is very far away. Thandie also had to move home to have better access to taxi services. Transport and distance issues in Madwaleni therefore have an impact on family structures when trying to attain better health access and further undermining family cohesiveness and integrity. Jaco’s experiences of childhood and growing up as an orphan included displacement with his adopted parent until the intervention of welfare services. Jaco was only able to access health services once a week, if required, when he could get a lift to town with the farmer’s wife. In an emergency, with no available ambulance vehicles, they phoned the police who sent a vehicle to transport him to the doctor in town rather than the local clinic. The same would also be true for a health care user in Madwaleni who is unlikely to be able to afford medical aid for payment for private medical care. These four case studies confirm that poverty tends to undermine affordability, availability and access to health services for some of the participants, as found in other studies.^25,27^

Both Madwaleni and Fraserburg are rural and remote. In Fraserburg, where there is no public transport, people have to rely on private transport and have to travel vast distances to secondary or tertiary/referral health facilities, making transport costly. In Madwaleni, Thandie can only use her wheelchair inside her home because of the hilly terrain which is not conducive to a wheelchair user. The vast distances and poor quality roads make access to health facilities more difficult. This is especially true for wheelchair users. Unlike Fraserburg, the other sites do have transport systems in place. In both Gugulethu and Madwaleni, despite costs being a barrier to using public transport, people with disabilities rely on transport because of crime in Gugulethu and the long distances and poor quality roads in Madwaleni.

In Madwaleni, the first taxi was not able to accommodate Thandie’s wheelchair and she needed to wait for another taxi which could accommodate both her and her wheelchair. Jaco, in Worcester, currently had no transport issues, but when he was living on a farm some distance from the nearest health facility, he relied on the farmer’s wife or the police services to transport him. This limited him and undermined his sense of freedom to access public health care services whenever he chose. In such instances, it appears that a comprehensive primary health approach is needed to address some of these challenges.^28,27^

## Systemic factors

Systemic factors have been referred to as factors in the existing health system that may hamper or facilitate people’s access to primary health care (PHC), such as the distribution of resources between different services, the emphasis on primary care and the extent of service integration.^8^ Most systemic factors affect all health care users and are not exclusive to vulnerable groups. However, some vulnerable groups, such as people with activity limitations, may be more affected by these factors.^5^

A major systemic factor illuminated in all four case studies and all four sites is the way health services are organised. This factor consists of many different components, some of which facilitate equitable access to health care, and some of which hamper it.

First, a largely facilitating factor is the transformation of the health services in SA, with a shift of emphasis and funding from large tertiary hospitals to PHC clinics and CHCs (National Department of Health, 1996). In all four study areas people’s first port of call for health care is their local clinic or CHC. The clinics or CHCs are located in the local communities and should therefore be much more available than the more centrally located hospitals. Despite this decentralisation of health services, access to local clinics and CHCs remains a challenge for many for a variety of reasons, as described in the previous section on structural and environmental factors. While these factors present barriers to all community members, their impact is perhaps greatest on people with activity limitations.^5^

The local clinics are all nurse-driven, with some often unreliable and inconsistent visiting doctor’s services. Xoliswa from Gugulethu was frustrated that access to secondary and tertiary services was gained through referral from primary level only. Furthermore, in Madwaleni and Fraserburg, both patients and staff complained of staff-shortages, leading to severely overworked staff, rapid staff-turnovers, long waiting times at the clinics and only limited time spent with each patient. These problems point to how public health services are organised and delivered. Mayosi et al.^29^ suggest that for South African public health care services to be effective more attention must be paid to issues of human resource development. This will assist the country to be on track with the mandate of the millennium development goals (MDGs) in delivering effective health services for the South African population.

Interestingly, in Worcester, a society particularly sensitised to disability issues, preferential treatment for people with disability is common. People with disability and people with chronic illnesses are often thought of as a group that is particularly affected by long waiting times, and are seen before the other patients, regardless of what their position in the queue. This approach was also common in Madwaleni.

To increase the efficiency of the health services, service delivery is often grouped into categories of health problems, such as TB, HIV, physiotherapy and so on. Services for a certain health problem are then offered at one or more set times throughout the week or month, and patients with that particular health problem must attend at that time. This is more efficient and suits many patients. However, if a patient misses the service on that particular day they may not have access to the service for another week or month or even longer. Furthermore, patients often have more than one health issue they would like to attend to.

PHC clinics are open for 8 hours a day, usually between 8 am and 4 pm. Emergencies that need to be tended to outside of the clinics’ opening hours have to be taken to the nearest health centre or hospital. In Madwaleni and Fraserburg these emergency services are located at a great distance. Thus, an emergency outside of the clinic’s opening hours may prove to be critical, or even fatal. This problem is not isolated to people with activity limitation.

While health services are free at primary level, access to secondary services usually incurs high transport costs. Specialist services such as dentistry, optometry and audiology are paid privately as these services are sometimes not available in public health care services. Many people with disabilities are dependent on specialist services on a regular basis; the lack of availability of these services in the public health care limits accessibility and hence makes people with limitation in activity more vulnerable to inequitable access to health care.

Similarly, many people with disabilities are dependent on medication that is only available at secondary or tertiary level of health care, and not at PHC clinics. Thus, people are either prescribed a less than optimal medication or are referred to a secondary or tertiary service hundreds of kilometres away. There is a policy which allows a special request for patients who need special medication to get this at clinic or CHC level.

Outreach services were limited in all four areas. In Worcester, there are mobile clinics; in Gugulethu, outreach is associated with special health care campaigns such as immunisations; and in Madwaleni, outreach services are sporadic and dependent on the availability of staff at the clinics and the hospital. There are community health workers providing some home-based care services, but these unskilled health workers are not allowed to carry any medication and are unable to do any testing or diagnostic work. In SA, there are policies that are aimed at assisting and guiding delivery of health care to the rural areas (National Department of Health, 1996). Despite this, participants from both Fraserburg and Madwaleni felt that district, provincial and national governments did not care about the health needs of their communities.

## Activity limitations

Across the world, affordable and accessible transport appears to be a major problem for people with activity limitations.^13^ In some of our more urban sites, wheelchair-dedicated public transport services are available but must be booked 7 days in advance and are not available when one experiences a sudden illness. There is a general reluctance to accommodate wheelchair users by local public transport, or extra charges are required, further burdening a person already struggling with poverty. For workers residing on farms, they are dependent on employers for transport to town. People close to health facilities may struggle to access health services because of crime, as people with mobility impairments are concerned about safety as they cannot run when attacked. Other areas such as Madwaleni have uneven terrain which are not suitable for a person with mobility impairments. Delays in accessing public health services increase vulnerability to secondary impairments.

While, in Worcester and Madwaleni, health professionals appear to be aware of the need to support people with activity limitations by allowing them to be seen first and not to wait in queues, they were found to be limited in the skills necessary to respond to the needs of individuals with impairments. Jaco experienced health service staff in Worcester as either unaware of his disability or unable to communicate effectively with him, even though his hearing impairment is indicated on his file. There is an urgent need for health professionals to learn how to communicate with patients with hearing impairments. Using sign language interpreters is not optimal as this threatens the deaf person’s constitutional right to privacy and confidentiality.^30^

People with activity limitations often do not have a choice of service provider or the option to ask for a second opinion. Poverty makes access to private health care impossible. Personal choice is also affected by availability and affordability of transport, ability to pay for private services and communication. As Jaco explains, you cannot access the services when you need them but have to wait until it is convenient for others. Despite the challenges experienced by people with activity limitations, some participants had positive things to say about health care providers and the health system. Jaco, despite trouble communicating, still feels that the doctors give him sufficient time during consultations, while Emily sees the nursing staff as showing positive, supportive attitude, very hard working and trusted by patients. Another positive aspect described by Emily is an appropriate suitable facility.

## Personal and cultural factors

Being disabled is still seen and interpreted negatively by many people.^31^ A majority of participants experienced lack of support, some from partners while for others from their family members. Lack of education undermines prospects for employment and locks individuals in a cycle of poverty. Social grants such as disability grants and child support grants bring some form of relief to people with activity limitations.^32,33^ Thandie is a single mother who mainly depends on a disability grant and child support grant for income. These grants barely meet her needs, nor do they meet the needs of people with activity limitations generally, and are insufficient for meeting needs such as emergency health care.

There is a constant interaction of personal incapacities, poverty, lack of social support, as well as a lack of educational opportunities and knowledge that further deepens an individual’s vulnerability status.^15,34^ Access to health services depends on the individual’s capacity to cope with these factors. Three of the participants (Xoliswa from Gugulethu, Jaco from Worcester and Thandie from Madwaleni) appeared to be struggling with the life challenges they face in accessing health services. Their vulnerability was because of poverty, distance and limitations of public transport. Despite their vulnerability, all the participants appeared to be confident and resilient enough to access public health care services independently when needed.

## Factors outside public health services limits users from critical appraising health services

We also observed from our case studies, especially those from the poverty stricken areas of Madwaleni and Gugulethu, that people focus more on factors outside the health service limiting their access, rather than problems within the health care system. Our participants did not highlight a lot of systemic factors, though they do seem to exist. Hence, there was a general lack of critical appraisal of health services. They experience such substantial problems with meeting their own basic needs that they are not in a position to even begin to evaluate the quality of care at the hospital and clinics. These barriers and facilitators beyond the public health service have implications for whether the client will attempt to access the public health service, and if they do, whether they will give up before they reach the public health services because of the barriers they come up against.

## Revisiting and adjusting the EquitAble conceptual framework

**FIGURE 2:**
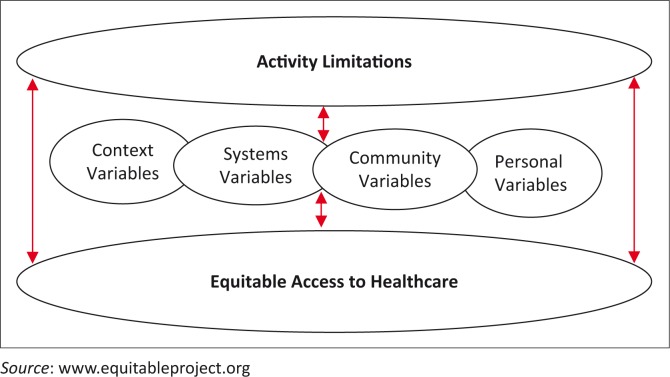
Adjusted EquitAble conceptual framework.

Earlier in this article, we presented a model based on the ICF and developed by the EquitAble project researchers. In Figure 2, these four case studies suggest a similar but adjusted model:

In this adjusted conceptual framework, we are suggesting that according to these four case studies, people with activity limitations interact with factors related to context, system, community and personal factors which influence their access to public health care services. These factors are interrelated and interwoven, acting as either barriers or facilitators to equitable access to health services for vulnerable groups such as people with activity limitations. Because of this interplay of barriers and facilitators experienced by an individual, a multifaceted approach is required to overcome the barriers faced by an individual in accessing public health services.

## Conclusions and recommendations

Similar to previous studies, the four case studies showed that factors that influence access to health services outside the health system are complex and interrelated, especially in poverty stricken areas.^2,13^ It is important to look beyond the health service at barriers and facilitators to explain access to public health facilities. Many problems experienced by people with activity limitations to accessing public health services may be the same, but the underlying factors may differ as we describe below.

Transport related factors might vary, presenting as a lack of transport, an inability to afford transport, attitudinal barriers of drivers who refuse to take the wheelchair on the bus/transport, long distances and bad road conditions, especially during rainy seasons in places such as Madwaleni. Understanding transport as a barrier to accessing health care requires a recognition of each related factor, and solutions required.^2^

Demographically, the four cases presented were only different from their peers because of their impairments and subsequent activity limitations they presented with. Even though their impairments were diverse, it was difficult to isolate one factor as the main cause of limited access to health services. For example, if activity limitation is compounded by lack of education, this results in a lack of employment opportunities further resulting in poverty.

Though public health has been made available and free for certain users such as people with activity limitations^35^, there are related costs such as transport, personal assistance and food as a visit to the public health centre may take the whole day. Participants in the four case studies experienced activity limitations that were compounded by varying degrees of poverty that made access to health services difficult.

Lack of choice regarding who to see in the public health service as well as not having access to the private health care system, because of lack of financial resources, appeared to leave the participants with a sense of helplessness, for example, helplessness experienced by clients with hearing disabilities who experienced significant communication difficulties within the health care system.^36^

These four case studies demonstrate that, while most problems are experienced by everyone, people with activity limitations are affected in particular ways making them particularly vulnerable. This implies that to address access for these participants, there is a need to address all four elements of PHC, that is: accessibility, affordability, equity and quality of public health services. This approach calls for the broad PHC strategy of 1978 that the South African government adopted in 1996.^28,37^ The revitalisation of PHC and the introduction of National Health Insurance (NHI) would open a window of opportunity for policy makers and policy implementers to revisit and address the areas of access to public health services for people with activity limitations.^27,29,38,39,40^ This article indicates the following areas that may require strengthening:

1. A coordinated public transport system that transports patients to the public health care centres at designated areas and times and back home once they have received health care services.
2. Training of health professionals in sign language to facilitate communication of health professionals with patients with hearing disabilities.
3. A focus on community level services beyond the clinic with more outreach programmes of specialist care directly addressing needs of people with activity limitations closest to their homes. It is in this regard that models of best practice such as community based rehabilitation might be useful strategies to use.

Other aspects not only targeting people with activity limitations but inclusive of all public health care users.

1. Workforce organisation and utilisation with clear referral pathways might also be necessary to ensure a smooth running of the health system.
2. Development of innovative models that link the South African private health care that is flourishing with the floundering public health care system.
3. A broad PHC strategy that addresses social problems such as crime, poverty and family cohesion is an urgent call for the revitalisation of PHC.
